# Indistinguishable NDM-producing *Escherichia coli* isolated from recreational waters, sewage, and a clinical specimen in Ireland, 2016 to 2017

**DOI:** 10.2807/1560-7917.ES.2017.22.15.30513

**Published:** 2017-04-13

**Authors:** Bláthnaid M Mahon, Carina Brehony, Elaine McGrath, James Killeen, Martin Cormican, Paul Hickey, Shane Keane, Belinda Hanahoe, Ann Dolan, Dearbháile Morris

**Affiliations:** 1 Antimicrobial Resistance and Microbial Ecology Group, School of Medicine, National University of Ireland, Galway, Ireland; 2 Carbapenemase-Producing Enterobacteriaceae Reference Laboratory, Department of Medical Microbiology, University Hospital Galway, Galway, Ireland; 3 Department of Medical Microbiology, University Hospital Galway, Galway, Ireland; 4 Environmental Health Service, HSE West, Galway, Ireland; 5 Galway County Council, Galway, Ireland

**Keywords:** antimicrobial resistance, carbapenemase-producing *Enterobacteriaceae*, New Delhi metallo-beta-lactamase, recreational waters, wastewater

## Abstract

In this study, New Delhi metallo-beta-lactamase (NDM)-producing *Enterobacteriaceae* were identified in Irish recreational waters and sewage. Indistinguishable NDM-producing *Escherichia coli* by pulsed-field gel electrophoresis were isolated from sewage, a fresh water stream and a human source. NDM-producing *Klebsiella pneumoniae* isolated from sewage and seawater in the same area were closely related to each other and to a human isolate. This raises concerns regarding the potential for sewage discharges to contribute to the spread of carbapenemase-producing *Enterobacteriaceae*.

We report the finding of New Delhi metallo-beta-lactamase (NDM)-producing *Enterobacteriaceae* in fresh water and seawater samples collected at two beaches located near an untreated human sewage ocean discharge. Isolates of NDM-producing *Escherichia coli* derived from the sewage collection system, the sewage storage tank and the outflow were 100% identical by pulsed-field gel electrophoresis (PFGE) to those derived from a fresh water stream on one of the beaches, and to a clinical isolate.

## Recreational water and sewage sample sites

**Figure 1:**
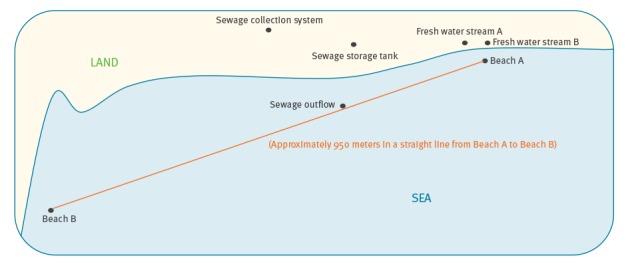
Schematic diagram of water and sewage sampling points and their location relative to each other, Ireland, 2016–2017

In 2016, we identified a beach (Beach A) in Ireland, used for bathing and recreation, which is crossed by two fresh water streams (Stream A and Stream B), flowing from the surrounding countryside. These streams were examined for the presence of carbapenemase-producing *Enterobacteriaceae* (CPE). The detection of NDM-producing *E. coli* in these waters prompted subsequent additional sampling of the streams. As untreated human sewage was being discharged into the sea in the vicinity of the beach, and the fresh water streams can become immersed in seawater at high tide, sewage was evaluated as a potential source. Sewage samples included samples from the collection system, the storage tank and the outflow. Sampling was performed in the period May to September, 2016. The sewage system is not linked to any hospital or long-term care facility that we are aware of. Further sampling of the fresh water streams and sewage sites was carried out in January 2017. In addition to this, seawater from Beach A and from a second beach (Beach B), ca 950 m in a direct line from Beach A were examined. Figure 1 shows a schematic diagram of the sampling points and their location relative to each other.

## Processing of samples

We applied a previously described method (CapE), to examine large volumes of water (30L) from both the fresh water streams and the seawater, for the presence of CPE [1]. Following filtration and overnight enrichment, the samples were sub-cultured onto Brilliance CRE agar (Oxoid). Sewage samples were examined by direct plating onto Brilliance CRE agar. Following purification, presumptive isolates were identified to species level by matrix-assisted laser desorption/ionisation time-of-flight (MALDI-TOF) mass spectrometry, and antimicrobial susceptibility testing was performed and interpreted in accordance with European Committee on Antimicrobial Susceptibility Testing (EUCAST) criteria [2]. Carbapenemase-encoding genes were detected by real-time PCR, as previously described [3-6]. Typing of NDM-producing *Enterobacteriaceae* was not performed at the variant level, but PFGE was performed on all isolates*,* as outlined previously [7]. PFGE profiles of NDM-producing *Enterobacteriaceae* isolated from recreational water and sewage samples were compared with PFGE profiles of NDM-producing *Enterobacteriaceae* isolated from clinical specimens.

## Findings of New Delhi metallo-beta-lactamase (NDM)-producing *Enterobacteriaceae*

**Figure 2:**
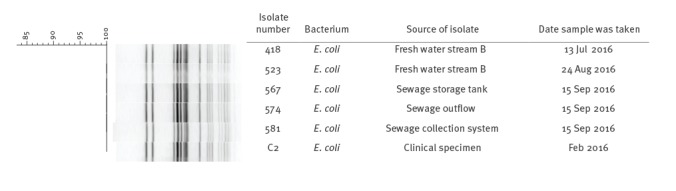
PFGE analysis of New Delhi metallo-beta-lactamase-producing *Escherichia coli* isolated from fresh water, sewage and a clinical source in Ireland, 2016–2017 (n = 6 isolates)

Of eight fresh water samples from Stream B, NDM-producing *E. coli* were isolated from two samples, which were collected on 13 July and 24 August 2016. NDM-producing *E. coli* were also isolated in samples collected on 15 September 2016 from the sewage collection system (one of two samples), the storage tank (one of two samples) and the sewage outflow (one of one sample) (Table). All isolates were resistant to ampicillin, cefotaxime, cefoxitin, cefpodoxime, ceftazidime, ciprofloxacin, ertapenem, meropenem and nalidixic acid. The isolates obtained from the fresh water and sewage samples are indistinguishable by PFGE analysis from a human isolate submitted to the National Carbapenemase Producing *Enterobacteriaceae* Reference Laboratory Service (CPERLS) in early 2016 (Figure 2).

**Table:** Overview of sampling sites, dates and detection of carbapenemase-producing *Enterobacteriaceae* in a coastal region in Ireland, 2016–2017

Sample site	Date of sampling	Carbapenemase-producing *Enterobacteriaceae*
**Fresh water Stream A**	25 May 2016	Not detected
	22 Jun 2016	Not detected
	13 Jul 2016	Not detected
	10 Aug 2016	Not detected
	24 Aug 2016	Not detected
	7 Sep 2016	Not detected
	15 Sep 2016	Not detected
	18 Jan 2017	Not detected
**Fresh water Stream B**	25 May 2016	Not detected
	22 Jun 2016	Not detected
	13 Jul 2016	NDM-producing *E. coli*
	10 Aug 2016	Not detected
	24 Aug 2016	NDM-producing *E. coli*
	7 Sep 2016	Not detected
	15 Sep 2016	Not detected
	18 Jan 2017	Not detected
**Sewage storage tank**	15 Sep 2016	NDM-producing *E. coli*
		NDM-producing *K. pneumoniae*
	18 Jan 2017	NDM-producing *K. pneumoniae*
**Sewage collection system**	15 Sep 2016	NDM-producing *E. coli*
		NDM-producing *K. pneumoniae*
	18 Jan 2017	NDM-producing *K. pneumoniae*
**Sewage outflow**	15 Sep 2016	NDM-producing *E. coli*
**Seawater Beach A**	18 Jan 2017	NDM-producing *K. pneumoniae*
**Seawater Beach B**	18 Jan 2017	NDM-producing *K. pneumoniae*

*E. coli*: *Escherichia coli*; *K. pneumoniae*: *Klebsiella pneumoniae*; NDM: New Delhi metallo-beta-lactamase.

PFGE: pulsed-field gel electrophoresis.

PFGE typing was performed with *Xba1*. All isolates are 100% similar by PFGE.

**Figure 3:**
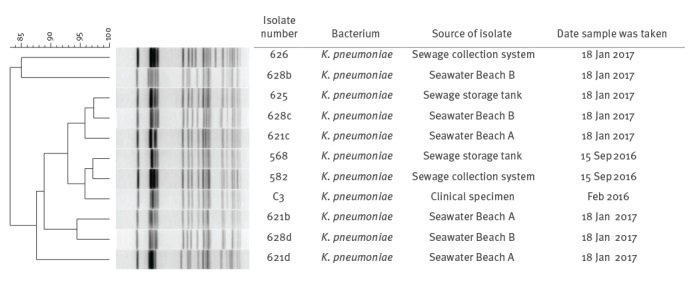
PFGE analysis of New Delhi metallo-beta-lactamase-producing *Klebsiella pneumoniae* isolated from seawater, sewage and a clinical source in Ireland, 2016–2017 (n = 11 isolates)

NDM-producing *K. pneumoniae* were isolated from two of three sewage sampling sites on 15 September 2016 and two of two sewage sampling sites on 18 January 2017. NDM-producing *K. pneumoniae* was also detected in seawater samples collected at Beach A and Beach B on 18 January 2017 (Table). These isolates were resistant to ampicillin, cefotaxime, cefoxitin, cefpodoxime, ceftazidime, ciprofloxacin, ertapenem, gentamicin, kanamycin, meropenem, nalidixic acid and tetracycline. PFGE analyses of isolates from sewage, seawater and a human isolate (from CPERLS) show isolates to be between 83% and 97% similar (Figure 3).

PFGE: pulsed-field gel electrophoresis.

PFGE typing was performed with *Xba1*. Isolates are between 83% and 97% similar by PFGE.

## Discussion

The rapid dissemination of carbapenemase-producing *Enterobacteriaceae* (CPE) in Europe and worldwide is making the delivery of effective healthcare an increasing challenge [8]. The number of CPE confirmed by the national reference laboratory in humans in Ireland has increased every year, rising from 48 in 2013, to 369 in 2016. In 2016, the three most commonly reported carbapenemases in Ireland were *K. pneumoniae* carbapenemase (KPC), carbapenem-hydrolysing oxacillinase-48 (OXA-48), and NDM [9]. The NDM gene is primarily plasmid-encoded, enabling its easy transfer between bacterial species. The plasmids are diverse and usually harbour a large number of other resistance genes [10]. The NDM gene has been detected extensively in the Indian subcontinent where it has been reported from both environmental and clinical sources [11]. Rapid global spread has been aided greatly by intercontinental travel [12]. However, a recent study in 2016 reported an outbreak of NDM-1-producing *Enterobacteriaceae* in a number of hospitals in Ireland, where links to foreign travel were not identified [13].

In Europe, a number of studies have reported the presence of CPE in recreational water, including Verona integron-encoded metallo-beta-lactamase (VIM) producing *K. pneumoniae* in a river in Switzerland in 2013 [14], KPC-producing *E. coli* in a river in Portugal in 2012 [15], VIM-1, VIM-34, and IMP-type metallo-beta-lactamase (IMP)-8 producing *E. coli* in the same Portuguese river in 2016 [16], and NDM-1 producing *K. pneumoniae* in the River Danube in Serbia, in 2016 [17]. Here we identify NDM-producing *Enterobacteriaceae* in environmental water samples collected at two adjacent beach sites in Ireland. As far as we are aware, this is the first such finding in bathing seawater in Europe. 

We consider that contamination of the environment with NDM-producing *Enterobacteriaceae* from the human sewage outflow is likely to be the source, and that the fresh water streams were contaminated by backwash of sewage onto the beach by tidal currents. The presence of NDM-producing *Enterobacteriaceae* in the bathing water (seawater) and at a separate bathing site ca 950 m in a direct line indicates the extent of this contamination. It is important to note that by the established regulatory standards, the bathing water quality in the area concerned has been consistently of sufficient quality [18]. Notwithstanding compliance with regulatory standards however, it is reasonable to conclude that those using a beach such as this for recreational purposes might be at least intermittently exposed to NDM-producing *Enterobacteriaceae*. Although, to date, there is no evidence that NDM-producing *Enterobacteriaceae* has been acquired as a result of exposure to this beach environment, Leonard et al. have recently reported on the level of risk of exposure to antibiotic resistant bacteria in coastal waters and its relationship to different types of water sports [19].

It appears therefore that there is potential for environmental contamination to contribute to a transition of CPE from largely healthcare-associated organisms, to organisms affecting the general population and the veterinary sector. From a public health perspective, the findings focus attention on the need to accelerate programmes to cease discharge of untreated sewage into the environment. This practice should be unacceptable in the context of discharges in the vicinity of popular bathing and recreation areas where human exposure is highly likely. 

We consider that our findings point to potential limitations of the use of *E. coli* as an indicator bacteria for bathing water quality based on the number of colony forming units (CFU) per 100 mL [20]. In our view, this approach does not reflect the pathogenicity of some variants of *E.* coli, such as Shiga-toxigenic *E. coli* for which the infectious dose is very low, (<10 CFU/mL) [21].
